# Effects of Photobiomodulation on Osteoarthritis from In Vivo and In Vitro Studies: A Narrative Review

**DOI:** 10.3390/ijms26188997

**Published:** 2025-09-16

**Authors:** Ryo Kunimatsu, Ayaka Nakatani, Shuzo Sakata, Kotaro Tanimoto

**Affiliations:** 1 Department of Orthodontics and Craniofacial Developmental Biology, Graduate School of Biomedical and Health Sciences, Hiroshima University, Hiroshima 734-8553, Japan; ryoukunimatu@hiroshima-u.ac.jp (R.K.); anakatan@hiroshima-u.ac.jp (A.N.); 2 Department of Orthodontics, Division of Oral Health and Development, Hiroshima University Hospital, Hiroshima 734-8553, Japan; shuzosakata@hiroshima-u.ac.jp

**Keywords:** osteoarthritis, photobiomodulation therapy, bone tissue, low-power laser therapy, joint disorder

## Abstract

Osteoarthritis (OA) is an inflammatory disorder characterized by metabolic changes in the bone tissue, including the degeneration of hyaline cartilage (articular cartilage) and fibrocartilage (including the meniscus and labrum), sclerosis of the subchondral bone, and osteophyte formation. OA poses a major challenge for adults of all ages, leading to increased morbidity and decreased quality of life. The current conventional therapies mainly focus on pain control, with no definitive or regenerative therapies to reverse OA progression available. Lasers consist of electromagnetic waves generated by radiation emitted by an excited material. In medicine and dentistry, photobiomodulation by low-power laser therapy (photobiomodulation therapy [PBMT]) has been widely applied clinically to promote healing, regenerate tissue, modulate inflammation, and relieve pain. Basic studies have explored the regulation of OA manifestations and joint inflammation using PBMT, as well as the mechanisms of action involved, and clinical research has validated the beneficial effects of PBMT for patients with OA. However, the effects of PBM on OA and its mechanisms of action remain unknown. Herein, we review basic research that has examined the effects of PBMT on OA using in vitro and in vivo testing and discuss future challenges and prospects.

## 1. Introduction

Osteoarthritis (OA) is a progressive and regressive joint disorder that is prevalent worldwide and poses a major challenge for adults of all ages, resulting in increased morbidity and reduced quality of life [1]. The Global Burden of Disease, an observational epidemiological study led by the World Health Organization [2,3], reported that, in 2020, 7.6% (~595 million people) of the global population was diagnosed with OA. In 2020, the global age-standardized prevalence was 8058.9 per 0.1 million women (5780.1 per 0.1 million men). The prevalence of OA is projected to increase by 132.2% over the next 30 years and by 60% to 100% by 2050 [2,3]. The current conventional therapies have focused mainly on pain control, and no definitive treatment to reverse the progression of OA is available. Despite effective interventions, including physiotherapy, intra-articular injections of hyaluronic acid, and joint replacement therapy, the underlying cause of OA is often not addressed [4,5,6]. The structural variations in OA include disorders involving articular cartilage, subchondral bone, ligaments, synovium, and periarticular muscles.

Articular cartilage is a hyaline cartilage composed of meshwork-like collagen type II, a cartilage matrix including proteoglycans, and moisture. When compressive forces are applied to the articular head during movement, these components play a cushioning role, conferring pressure tolerance to the articular head. The cells in the cartilaginous tissue are primarily surrounded by an extracellular matrix (ECM) consisting mainly of chondrocytes, which provide collagen and proteoglycans, which in turn play a role in repairing damaged cartilage [7]. As no blood or lymphatic vessels are present in the cartilaginous tissue, nutrition is provided via simple diffusion from the synovial fluid through a concentration gradient. Therefore, the proliferative activity and tissue repair capacities of chondrocytes are low, and pathologies, including cartilage degeneration, are susceptible to subclinical progression [8].

Temporomandibular joint-OA (TMJ-OA) is characterized by regressive changes in the TMJ and has a complex pathology confounded by various factors, including the inflammatory response, mechanical loading, and disruption of cartilaginous tissue [9]. The prevalence of TMJ-OA increases with age, and clinical presentations on imaging include cortical bone tears (erosion), osteophytes (osteophyte), osteosclerosis of the mandibular head (generalized sclerosis), atrophic mandibular head (atrophy), and subchondral cysts (subchondral cyst). Excessive mechanical loading, given the absence of articular disc cushioning, has been considered a pathogenetic mechanism of TMJ-OA since individuals with TMJ-OA exhibit non-repositioning articular disc transposition. Although the pathogenesis of TMJ-OA has been investigated, a detailed understanding is lacking [10].

TMJ-OA exacerbation is a consequence of excessive mechanical loading applied to the mandibular head, which promotes cartilage matrix disruption via increased production of proinflammatory cytokines, including interleukin (IL)-1β and matrix metalloproteinase (MMP), as well as increased frictional factors, on articular cartilage surfaces [11]. Treatment of TMJ-OA includes surgical and non-surgical treatments, of which the most common modalities involve wearing splints and administration of non-steroidal anti-inflammatory drugs (NSAIDs) [12]. Splints reduce excessive mechanical stress applied to the joint by temporarily preventing overloading of the TMJ by involuntary movements during sleep. NSAIDs, including ibuprofen, reduce pain and relieve inflammation in TMJ-OA. Although both treatments are effective at inhibiting TMJ-OA progression and relieving the condition, they are symptomatic treatments that do not address the underlying causes.

Photobiomodulation therapy (PBMT) has been reported to improve the pathology of OA in articular cartilage [13]. PBMT is a therapeutic approach in which low-power exposure to red or near-infrared (NIR) spectral-domain lasers or light-emitting diodes (LEDs) increases cellular activity at the irradiated site without inducing thermal injury to the tissue [14]. Light energy in the red or NIR regions is absorbed by cytochrome C oxidase in the mitochondrial respiratory chain, which activates the electron transport chain, leading to various cellular events via second messengers, including reactive oxygen species (ROS), ATP, cyclic adenosine monophosphate, nitric oxide (NO), and calcium ions [15]. Light waves in the red or NIR spectral domain are expected to have clinical efficacy in deeper subcutaneous tissue owing to higher energy reaching deeper tissue compared to other visible or ultraviolet spectral domains [16]. PBMT has been reported to be effective in wound healing [17], bone regeneration [18], pain inhibition [19], reducting edema [20], and improved TMJ motor function [21], indicating its potential for use in new treatment strategies for TMJ-OA. However, the specific effects of PBMT on cells and tissues are still under investigation, and the associated efficacy, safety, and mechanisms of action require further validation.

Herein, we review those studies that evaluated the effects of PBMT on chondrocytes and articular tissue. Our primary aim was to summarize the current knowledge and concerns and to discuss future research directions for developing effective treatments.

## 2. Search Strategy and Study Selection

We searched PubMed to retrieve relevant articles published between 1994 and 2024 using the following terms “laser OR LLLT OR Photobiomodulation” AND “chondrocyte OR osteoarthritis OR OA OR cartilage OR TMJ-OA”; “Mesenchymal Stem Cell OR MSC OR stem cell” AND “laser OR LLLT OR Photobiomodulation” AND “chondrocyte OR osteoarthritis OR OA OR cartilage OR TMJ-OA”. The inclusion criteria were original review articles published in English. We excluded papers that were not published in English or were not considered relevant based on their titles and abstracts. Next, full-text reviews were performed to further exclude articles that did not fall within the scope of this review.

Relevant publications were retrieved from the reference list and further analyzed to determine whether they met the inclusion criteria. The authors retrieved data, but the quality and bias of the retrieved articles were not interpreted.

## 3. Effects of PBMT on OA Studies In Vivo

The effects of PBMT on OA reported in the literature are presented in Table 1. First, many animal experiments have demonstrated the effect of PBMT on suppressing pain in OA joints and improving joint movement function. The methods for producing animal OA models are roughly divided into chemical methods, in which a drug is administered to the joint, and surgical methods. In most studies, chemical methods are used to establish models of OA-induced pain.

Monoidoacetate (MIA) is an inhibitor of glyceraldehyde-3-phosphate dehydrogenase that inhibits cellular glycolysis [22]. MIA has been widely used to establish rodent models of OA-induced pain because its intra-articular injection causes chondrocyte death, cartilage degeneration, and subsequent changes in subchondral bone, including osteophyte formation [22]. Micheli et al. [23] reported the inhibitory effects of PBMT with an NIR laser (905 nm, 0.32 J/cm^2^) on 2 mg/25 μL MIA-induced pain in a rat tibiotarsal joint. Mechanistically, this result suggests that PBMT not only suppresses the production of inflammatory cytokines but also exerts a direct effect on neuronal cells, resulting in increased pain thresholds.

Yamada et al. [24] suggested that the oxidative stress-related mechanism may be involved in the suppression of MIA-induced pain by PBMT. The authors induced OA by administering 1.5 mg/50 μL of MIA intraarticularly in rat knee joints. The increased hyperalgesia caused by MIA was suppressed by PBMT with a GaAl laser (904 nm, 6 or 18 J/cm^2^). At the same time, IL-1β, IL-6, and TNF-α induced by MIA administration were significantly suppressed by PBMT at 18 J/cm^2^. Furthermore, superoxide dismutase, an enzyme that suppresses ROS induction, was observed following PBMT administration. Oxidative stress, which involves an imbalance between ROS production and antioxidant capacity, plays a key role in the development of OA. These findings indicate that PBMT may reduce joint pain through suppressing OA cartilaginous inflammation and increasing the antioxidant capacity of the organism.

PBMT may reduce NO levels along with cytokines, thereby reducing pain in OA joints [25]. MIA was administered into the knee joint of rats, followed by PBMT (904 nm, 18 J/cm^2^) and 10 mg/kg sodium diclofenac (an NSAID of the phenylacetic acid family) to reduce pain sensation. The biochemical analyses, including the measurement of lipid peroxidation, protein carbonyl content, non-protein thiol levels, superoxide dismutase activity, and NO levels, revealed a reduction in NO, proinflammatory cytokine, and oxidative stress-induced injury levels. Furthermore, Balbinot et al. [26] reported that PBMT improved joint function. OA was induced by administering a single dose of 2 mg/50 μL MIA into the knee joint space of rats. PBMT with a GaAlA diode laser (850 nm, 57.14 J/cm^2^) improved disturbance to joint movement.

Other researchers have established OA models through intra-articular administration of papain [27,28,29]. Papain, a proteolytic enzyme, degrades the protein–polysaccharide complex of the articular cartilage, which releases chondroitin sulfate and proinflammatory cytokines (TNF-α and IL-1β) as well as MMP and free radical products [27]. In a model with OA induced using papain, OA-like joint destruction is likely to occur through arthritis. Further, de Oliveira et al. [28] induced OA by injecting 20 μL of 4% papain into the knee joint cavity of rats. Immediately following injection, PBMT was applied with a GaAlAs diode laser (808 nm, 144 J/cm^2^), and the hyperalgesia caused by papain was improved. In addition, levels of TNF-α, cytokine-induced neutrophil chemoattractant-1 (CINC-1), and bradykinin receptors B1 and B2 were elevated in the papain-induced OA joints and were significantly suppressed by PBMT. In this OA model, PBMT may raise the pain threshold by reducing the expression of bradykinin receptors in addition to regulating the expression of proinflammatory cytokines.

**Table 1:** Summary of the effects of PBMT on OA models in in vivo studies.

Study ID	Author	Year	Irradiation Conditions	Animal OA Model	Results
[23]	Micheli et al.	2017	808 nm, 905 nm Pulsed 125–1940 mW 0.16–1.08 J/cm^2^ 4–124 s 10–13 sessions	Rat MIA, CFA	PBMT ameliorated the mechanical hyperalgesia induced by MIA and CFA.
[24]	Yamada et al.	2020	904 nm Pulsed 40 mW 6, 18 J/cm^2^ 54 s 8 sessions	Rat MIA	PBMT reduced oxidative stress and suppressed mechanical hyperalgesia in joints, sera, and spinal cord and IL-1β, IL-6, and TNF-α expression.
[25]	Yamada et al.	2022	904 nm Pulsed 40 mW 70 W (Peak power) 50–500 J/cm^2^ 10–100 s 8 session	Rat MIA	PBMT reduced pain sensation and oxidative stress-induced injury, NO levels, and inflammatory cytokines and alleviated oxidative stress in sites distant from the lesion.
[26]	Balbinot et al.	2021	850 nm Continuous 100 mW 57.14 J/cm^2^ × 4 40 s × 4 15 sessions	Rat MIA	PBMT ameliorated hyperalgesia and motor deficits, suppressed extracellular matrix disruption in articular cartilage, and reduced the number of glial cells in the spinal dorsal horn.
[28]	de Oliveira et al.	2017	808 nm 50 mW 144 J/cm^2^ 80 s	Rat Papain	PBMT ameliorated hyperalgesia and suppressed the expression of TNF-α, CINC-1, and bradykinin receptors B1 and B2.
[29]	Alves et al.	2014	808 nm Continuous 50 and 100 mW 142 J/cm^2^ 40 or 80 s 4, 7, and 10 session	Rat Papain	PBMT reduced collagen type III expression and increased collagen type I expression. LLLT at 50 mW significantly reduced MMP-2 expression at 21 days compared with the injured group. LLLT at 50 mW significantly reduced MMP-9 expression at 21 days compared to LLLT at 100 mW.
[30]	Tamazoni et al.	2017	830 nm Continuous 100 mW 214.2 J/cm^2^ 60 s	Rat Papain	PBMT suppressed IL-1β, IL-6, TNF-α, and PGE2 expression.
[31]	Tomazoni et al.	2016	830 nm Continuous 100 mW 214.2 J/cm^2^ 60 s 3 times per week 24 session	Rat Papain	PBMT and NSAID administration reduced the total number of cells in the inflammatory infiltrate and *MMP3* expression. PBMT strongly repressed the expression of myeloperoxidase involved in joint degradation and *MMP13* expression.
[32]	dos Anjos et al.	2022	830 nm Continuous 10 mW 3, 30 J/cm^2^ 15, 150 s 4 sessions	Mouse Zymosan	PBMT suppressed MMP-2, MMP-9, MMP-13, and MMP-14 expression and promoted TIMP-2 expression.
[33]	Lemos et al.	2016	830 nm Continuous 30 mW 3 J/cm^2^ 12 s 7 sessions	Rat CFA	PBMT prevented joint disc thinning and collagenous fibril and glycosaminoglycan reduction and reduced MMP-2 and MMP-9 activity.
[34]	Pallotta et al.	2012	810 nm Continuous 100 mW 50–500 J/cm^2^ 10–100 s 1 session	Rat Kaolin Carrageenan	PBMT decreased white blood cell count, myeloperoxidase activity, IL-1 and IL-6 expression, and prostaglandin E2 levels in joint lavage fluid.
[35]	Wang et al.	2014	830 nm (He–Ne) Continuous 50 mW 4.8 J/cm^2^ 300 s 3 times per week	Rabbit Anterior cruciate ligament excision	PBMT relieved knee-related pain and reduced synovial inflammation. IL-1β, iNOS, and MMP-3 expression was significantly reduced at 6 weeks. After 8 weeks, PBMT significantly reduced IL-1β, iNOS, MMP-1, MMP-3, and MMP-13 expression. PBMT increased collagen-2, aggrecan, and TIMP-1.
[36]	Assis et al.	2016	808 nm 50 mW 50 J/cm^2^ 28 s 24 sessions	Rat Anterior cruciate ligament excision	PBMT improved OARSI scoring of osteoarthritis and suppressed IL-1β, caspase-3, and MMP-13 expression.
[37]	Assis et al.	2018	808 nm Continuous 50 mW 50 J/cm^2^ 28 s 24 sessions	Rat Anterior cruciate ligament excision	PBMT reduced OA grading of joints and promoted IL-10 and collagen II expression.
[38]	Trevisan et al.	2020	850 nm Continuous 200 mW 12 J/cm^2^ 30 s 12 sessions	Rat Anterior cruciate ligament excision	PBMT improved joint OARSI scoring and promoted type II collagen and TGF-β expression.
[39]	Sanches et al.	2018	808 nm Continuous 50 mW 50 J/cm^2^ 1.7 W/cm^2^ 28 s 3 times per week 29 session	Rat Anterior cruciate ligament excision	OARSI score for cartilage degeneration was significantly higher for control than for CS/Gl and CS/Gl + PBMT groups. CS/GS + PBM decreased IL-1β expression and increased IL-10 and Col II immunoexpression.
[13]	Xiang et al.	2020	Systematic review and meta-analysis		PBMT positively affected cartilage defects in animal knee models under proper irradiance and adequate irradiation time.
[40]	Nambi	2021	Systematic review		PBMT was associated with mild to moderate decreases in IL-1β, TNF-α, and MMP-13 expression, which provided inflammatory relief. IL-6 was not reduced.

CFA, complete Freund’s adjuvant; CS/Gl, chondroitin sulfate and glucosamine sulfate; MIA, monoidoacetate; NO, nitric oxide; OA, osteoarthritis; OARSI, Osteoarthritis Research Society International; PBMT, photobiomodulation therapy.

PBMT not only reduces the expression of inflammatory cytokines but also affects cartilage matrix metabolism. Alves et al. [29] generated a rat model with knee joint OA induced by papain injection, and PBMT was applied with an AsGaAl laser (808 nm, 142 J/cm^2^). PBMT at 50 mW and 100 mW decreased type III collagen and increased type I collagen, resulting in cartilaginous tissue repair. Interestingly, PBMT was more effective at 50 mW than at 100 mW for suppressing MMP-2 and -9. Similarly, PBMT (830 nm, 214.2 J/cm^2^) resulted in decreased gene expression of *IL1B* and *IL6*, protein expression of TNF-α, and plasma levels of prostaglandin E2 (PGE2) in a rat model of papain-induced OA in knee joints, demonstrating that its anti-inflammatory effect was greater than that of local administration of NSAIDs [30].

The anti-inflammatory effects of PBMT on cartilage matrix metabolism were found to be more effective in a papain-induced OA model when combined with other therapies. Tomazoni et al. [31] compared the efficacy of PBMT (830 nm, 214.2 J/cm^2^) and topical NSAID when applied alone or in combination in a rat model of papain-induced OA in knee joints. The combination of PBMT and NSAID significantly reduced the total number of inflammatory infiltrates and significantly decreased *MMP3* gene expression in cartilage tissue. However, studies on the combination of PBMT with other therapies, such as medication or exercise therapy, are lacking, and future developments in this field are anticipated.

The effects of PBMT have also been examined experimentally by administering drugs such as zymosan [32], complete Freund’s adjuvant [33], and kaolin and carrageenan [34] intraarticularly. However, these were aimed at inducing relatively severe inflammation in the joints and therefore were not further considered in this review as they were not considered to be OA models.

One surgical method for establishing OA models involves removing the anterior cruciate ligament (ACL) and increasing the mechanical load on the joint. Compared to administering drugs to the joint, this method does not require consideration of the secondary effects of drugs and can induce OA in a more natural manner. However, it is difficult to stabilize the condition because the surgical procedure is influenced by the surgeon.

Wang et al. [35] observed daily changes in a rabbit progressive OA model induced via ACL transection treated with He–Ne laser irradiation (830 nm, 4.8 J/cm^2^) three times weekly for 2, 4, 6, and 8 weeks. Pain assessment and histological evaluation of synovitis showed that laser irradiation relieved knee pain after 6 weeks and reduced synovial inflammation. In cartilaginous tissues, compared with the non-irradiated group, laser irradiation significantly reduced *IL1B*, *NOS2*, and *MMP3* expression at 6 weeks and further reduced *IL1B*, *NOS2*, *MMP1*, *MMP3*, and *MMP13* expression at 8 weeks but significantly increased type II collagen, aggrecan, and TIMP-1 levels.

Assis et al. [36] exposed a rat model of knee OA to GaAlA diode laser irradiation (808 nm, 50 J/cm^2^) three times weekly for 8 weeks starting 4 weeks after the resection of the ACL. PBMT improved Osteoarthritis Research Society International (OARSI) scores, an index of the severity of OA, and significantly suppressed the elevated expression of IL-1β, caspase-3, and MMP-13 in the articular cartilage. In another study from the same group [37], PBMT resulted in decreased OA grading and increased expression of the anti-inflammatory cytokine IL-10 and type II collagen. This study also examined the effectiveness of PBMT combined with exercise therapy, and no differences were observed as compared with PBMT alone. These studies suggest that PBMT may exert anti-inflammatory, analgesic, and cartilage-reparative effects on rat models of OA induced through ACL resection.

Trevisan et al. [38] established a rat model of OA induced through ACL resection followed by irradiation with GaAlA LED (850 nm, 12 J/cm^2^) three times weekly for 4 weeks starting 4 weeks after the operation. PBMT resulted in decreased OARSI scores with increased expression of type II collagen and TGF-β. These findings suggest that PBMT with exposure to LED may exhibit a reparative effect on articular cartilage by enhancing the synthesis of type II collagen, a major component of the cartilage matrix, as well as the induction of TGF-β.

Sanches et al. [39] proposed a combination therapy for cartilage tissue repair. They investigated the efficacy of PBMT combined with the administration of chondroitin sulfate and glucosamine sulfate (CS/Gl), a cartilaginous matrix constituent, in achieving inflammatory and reparative effects in a rat knee OA model induced through ACL transection. The OARSI scores significantly decreased following both CS/Gl administration alone and CS/Gl + PBMT in the OA model group. In particular, decreased IL-1β and increased IL-10 and type II collagen expression were observed in the CS/GS + PBMT group. These findings suggest that the administration of CS/Gl combined with PBMT may effectively modulate inflammation and prevent the degradation of articular cartilage.

Several meta-analyses have also been reported [13,40]. Xiang et al. [13] conducted a meta-analysis to review evidence on the effectiveness of PBMT in treating cartilage defects in animal models of knee arthritis. Fourteen articles were extracted, and the results on microscopic morphological changes in animal models supported the efficacy of PBMT for cartilage restoration. Another systematic meta-analysis evaluated the effects of PBMT on inflammatory cytokines in a rat OA model [40]. Eight articles were extracted, all of which described studies that applied a GaAlAs laser (780–830 nm, 20–100 mW, 10–214 J/cm^2^). PBMT resulted in mild to moderate decreases in IL-1β, TNF-α, and MMP-13 protein expression, but not IL-6, leading to inflammatory relief. Therefore, PBMT may impact the protection of cartilage and the severity of cartilage pathology.

Taken together, the evidence presented suggests that PBMT suppresses the inflammatory state observed in OA and halts OA progression by suppressing the expression of inflammatory cytokines and modulating the expression of MMPs. However, the appropriate ranges of laser wavelength, irradiation dose, and irradiation time for cartilage repair remain unclear, indicating the need for further investigation to clarify the optimal conditions [13].

## 4. Effects of PBMT on Articular Cartilage-Related Cells In Vitro

Compared with assessments using in vivo investigations, those using in vitro experimental systems are scarce (Table 2). In particular, studies on PBMT have focused on fibroblasts, whereas studies using chondrocytes are limited. However, to fully understand the effect of PBMT on OA, it is essential to elucidate the molecular mechanisms using in vitro experimental systems.

In vitro investigations have suggested that PBMT suppresses the expression of several inflammatory cytokines and MMPs and enhances the production of type II collagen and aggrecan [41,42,43,44,45]. Hang et al. [43] suggested that laser irradiation (830 nm, 0.056 W, 5 J/cm^2^, once every 2 days) significantly increased type II collagen expression but decreased type I collagen expression in cultured rabbit chondrocytes. Yang et al. [42] reported that He–Ne laser irradiation (632.8 nm, 5.74 J/cm^2^) in cultured primary rabbit chondrocytes significantly increased chondrocyte synthesis of the ECM and enhanced the expression of type II collagen, aggrecan, and *Sox9*. Laser irradiation also suppressed the IL-1β-induced expression of ADAMTS5, MMP-13, and TNF-α, suggesting that PBMT suppresses the production of cartilage matrix degradation enzymes.

Signaling pathways involved in the effects of PBMT have been suggested. Chen et al. [41] applied oxidative stress to cultured primary porcine chondrocytes with 100 μM of H_2_O_2_. Through prophylactic irradiation with 635 nm LED, H_2_O_2_-induced free radical production was significantly suppressed, as was the gene expression of *IL1B* and *TNF*. The anti-inflammatory effects of LED irradiation were strengthened with antioxidants, vitamin E, or resveratrol, and a possible contribution of NF-κB, a transcription factor that combines inflammation-inducing and anti-inflammatory effects, was suggested.

High-frequency NIR semiconductor laser irradiation has also been reported [44]. This laser emits pulsed waves at minute frequencies, allowing light energy to penetrate tissue more efficiently with high peak output while avoiding thermal damage to the irradiated tissue. This type of irradiation (910 nm, 8 J/cm^2^) significantly suppressed IL-1β-induced MMP-1 and -3 accompanied by inhibition of *IL6* and *TNF* gene expression in cultured human primary knee articular chondrocytes [44]. Chen et al. [41] also suggested the involvement of a similar signal transduction pathway in their study using a high-frequency NIR semiconductor laser. Furthermore, this type of irradiation suppressed the increased phosphorylation, nuclear translocation, and transcriptional activity of NF-kB in chondrocytes treated with IL-1β [45]. These findings indicate that the activity of NF-kB, a transcription factor that plays a major role in OA pathogenesis, can be controlled by high-frequency NIR semiconductor laser irradiation.

Cytological examinations have clarified the cellular response and transmission route of PBMT. However, the involvement of various cytokines and complex signaling pathways has been inferred from reports investigating PBMT. Therefore, the detailed transmission pathways for PBMT need to be elucidated. Furthermore, in vitro studies have suggested that PBMT enhances the proliferation of chondrocytes [42,46,47,48,49]. Tim et al. [42] reported that GaAlA diode laser irradiation (808 nm, 28 or 50 J/cm^2^) induced chondrocyte proliferation in cultured primary rat chondrocytes. Anbari et al. [48] performed semiconductor laser irradiation (808 nm, 1, 2, 3, 4, and 5 J/cm^2^) on human chondrocytes and reported that irradiation below 5 J/cm^2^ did not significantly affect chondrocyte proliferation, whereas irradiation > 4 J/cm^2^ enhanced chondrocyte proliferation.

Regarding the frequency, Torricelli et al. [47] performed GaAlA diode laser irradiation (780 nm, 2500 mW) on rabbit and human chondrocytes for 5 days to evaluate cell proliferation. Irradiation at both 100 and 300 Hz was significantly effective. Furthermore, He–Ne laser irradiation (632.8 nm, 5.74 J/cm^2^) significantly enhanced cell viability through suppression of IL-1β-induced caspase-3 and Fas-associated death domain protein (FADD) in cultured primary rabbit chondrocytes [42].

## 5. Effect of Mesenchymal Stem Cells with PBMT on OA

Regenerative medicine using stem cell transplantation has shown promise for the treatment of OA. Mesenchymal stem cells (MSCs) were first identified as colony-forming cells with the capacity to differentiate into osteoblasts, adipocytes, and chondrocytes within myeloid organs [50]. As MSCs can be harvested from tissues and grown under standardized culture conditions, they are used as a transplanted cell preparation for autologous transplantation in the medical field, and cell preparations for the regeneration of skin and cartilage are already available on the market [51,52]. Studies have evaluated the potential of MSCs for cartilaginous tissue regeneration in vitro and in vivo [53]. Clinical trials have demonstrated that MSCs obtained from bone marrow, adipose tissue, and cord blood may be efficacious in the treatment of OA [53]. Thus, research has promoted the practical application of MSCs for the regenerative treatment of OA tissue. Below, we summarize the effects of stem cell transplantation and PBMT combination therapy on OA (Table 3).

Stancker et al. [54] examined the effect of intra-articular injections of adipose-derived stem cells (ADSCs) combined with PBMT (808 nm, 71.2 J/cm^2^) in a rat OA model with papain-induced inflammation. The combined treatment prevented degenerative modifications of COL-2-1 and reduced cytokine and MMP levels after 7 days. Tanideh et al. [55] established a guinea pig model of OA by resecting the ACL, which they treated with a combination of ADSC and two-wavelength (808 and 405 nm, 0.5 or 1 W, daily for the first week, followed by laser irradiation 4 times in week 2, twice in week 3, and once in weeks 4 and 5) laser irradiation to the knee. The combination of ADSC and PBMT improved cartilage-, surface-, matrix-, space-width-, osteophytes-, and radiological OA scoring more effectively than the respective methods alone.

Stancker et al. [54] suggested that ADSCs indirectly stimulate the secretion of bioactive factors, such as cytokines and growth factors, and that there are two potential mechanisms underlying the efficacy of stemness in the treatment of OA. First, the transplanted cells differentiate into chondrocytes and fill the cartilage lesion. Second, paracrine signaling and the secretion of various soluble and insoluble proteins affect the microenvironment. Regarding the mechanism of PBMT, it enhances cellular responsiveness in terms of gene expression, secretion of growth factors, and cell growth through increased mitochondrial membrane potential and increased ATP and cAMP levels. PBMT also increases the number of early stem cells prior to differentiation and the number of differentiated cells during tissue engineering and regenerative and healing processes. In addition, PBM induces biological modifiers.

In contrast, Tanideh et al. [55] reported that PBMT likely affects osteoblast differentiation and mineralization of myeloid MSC via the Wnt/β-catenin signaling pathway. In addition, increased expression of TGF-beta-gene has been observed after PBMT, highlighting its roles in regulating joint wellbeing by improving aggrecan and collagen synthesis and inhibiting degenerative mediators and enzymes, such as IL-1, collagenase, and stromelysin.

However, the molecular interactions of both therapies remain unclear. Further research is needed to understand the molecular interactions of both therapies in the presence of degenerative joint disease.

El-Qashty et al. [56] reported that combining a conditioned medium of ADSCs with PBMT (38 J/cm^2^) efficiently promoted arthritis healing, as did combining ADSC transplantation with PBMT. Furthermore, TGF-β expression is increased following PBMT, suggesting that it may play a role in regulating joint well-being by improving aggrecan and collagen synthesis and inhibiting degenerative mediators and enzymes, including IL-1, collagenase, and stromelysin [57,58,59,60]. Several mechanisms have been proposed for how PBMT affects MSCs, including modulation of paracrine activity, promotion of chondrocyte histogenesis, and promotion of subchondral bone microarchitecture [61]. PBMT may affect osteogenic differentiation and mineralization of myeloid MSCs via the Wnt/β-catenin-signaling pathway [62,63]. The role of PBMT in promoting MSC growth and differentiation has been previously demonstrated [53]. Tanideh et al. [55] discussed that the in vitro study by Jin et al. identified that BM-MSC-derived exosomes maintain chondrocyte wellbeing by promoting the synthesis of type II collagens and inhibiting IL-1-induced senescence and apoptosis. Also, the mechanism of certain exosome lncRNA(MEG-3) contributes to the anti-OA efficacy. However, some limitations exist, such as the wide variety of unknown components that constitute the secretome, the need for standardization of secretome production, and the variable factors that influence secretome composition, such as passage number and culture conditions. Further investigations and research on all these issues are required before the stem cell secretome can be widely applied as an effective treatment for OA.

Karic et al. [64] suggested that cell viability and ATP proliferation increased after 72 h of laser irradiation (660 nm, 5 J/cm^2^). In addition, flow cytometry at 1 and 2 weeks post-irradiation and immunofluorescence provided evidence for the differentiation of ADSCs into fibroblastic and chondrogenic phenotypes. This study highlighted the potential to promote cartilaginous regenerative repair by combining MSC implantation with laser irradiation. However, further in-depth examination is required. Although MSCs can be obtained from various sources and exhibit diverse properties, they can only be harvested in relatively low numbers and have poor growth capacity in vitro, making it difficult to secure enough cells for transplantation. These shortcomings may hamper the use of MSCs in clinical trials and practical applications. According to reported findings, PBMT has the potential to improve regenerative therapy by ensuring a sufficient number of MSCs for transplantation and providing an optimal milieu for MSCs prior to transplantation. Future studies should carefully evaluate the efficacy of PBMT in various types of MSCs and elucidate the optimal PBMT protocol.

Treating arthritis by focusing on the anti-inflammatory effects of MSCs has also received much attention in recent years [65]. Non-genetically modified MSCs (Edu-MSCs) can effectively treat inflammation-related diseases, including RA and OA. To enhance the limited anti-inflammatory activity of MSCs, gold nanostars loaded with triamcinolone bound to MSCs (Edu-MSCs-AuS-TA) not only prevented the progression of arthritis in moderate arthritis models but also restored advanced arthritis when used in conjunction with NIR laser irradiation. Edu-MCS/AuS-TA combined with PBMT effectively reduced advanced arthritis, which cannot be controlled by Edu-MSCs-AuS-TA alone [65]. Thus, combining non-genetic MSCs with laser irradiation may provide an effective strategy for indirectly treating inflammation. However, further studies are needed to confirm this hypothesis. Furthermore, future studies should aim to elucidate the molecular interactions between treatments in the presence of degenerative joint disease, and their relevance to other factors should be extensively studied.

**Table 3:** Effects of MSCs combined with PBMT for OA.

Study ID	Author	Year	Irradiation Conditions	Animal OA Model	Results
[54]	Stancker et al.	2018	808 nm Continuous 50 mW 71.2 J/cm^2^ 40 s Daily for 7 days	Rat Papain	Combined intra-articular injection of PBMT and ADSCs prevented degenerative modification of COL2-1 and reduced cytokines and MMPs.
[55]	Tanideh et al.	2024	808 nm, 405 nm Continuous 0.5 or 1 W 0.5 J/cm^2^ 12 min 15 sessions	Guinea pig Anterior cruciate ligament excision	ADSCs or PBMT alone results in good radiological and histological indices. ADSC and PBMT combined improved radiological OA scoring more effectively than either method alone.
[56]	El-Qashty et al.	2023	980 nm Continuous 0.5 W 38 J/cm^2^ 60 s Every 48 h for 7 days 4 sections	Rat TMJ Complete Freund’s adjuvant injection	ADSCs + LLLT and ADSCs-CM + LLLT restored joint structure with normal cartilage and disc thickness. Inflammation was significantly suppressed based on the significant reduction in TNF-α-positive immunostaining compared to the arthritic group. Cartilage proteoglycan content also significantly increased relative to that in the arthritic group.

ADSC, adipose-derived stem cell; MSC, mesenchymal stem cell; TMJ, temporomandibular joint.

## 6. Conclusions

**Figure 1:**
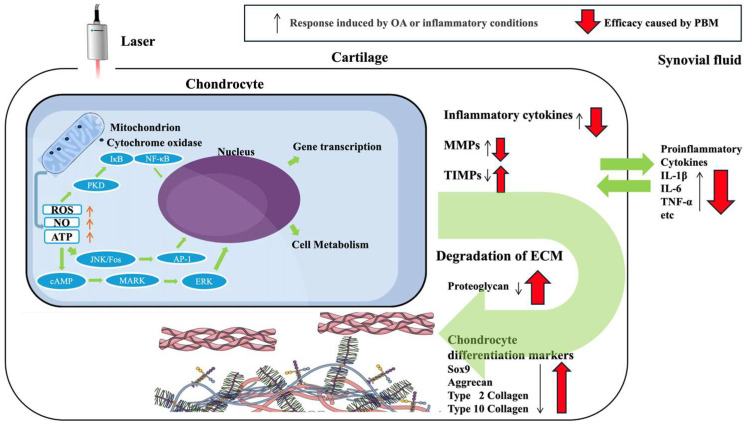
The summary of this review, and the effect of PBM on OA and a set of possible mechanisms. MMPs, matrix metalloproteinase; TIMPs, Tissue Inhibitors of Metalloproteinases; ECM, extracellular matrix; ROS, reactive oxygen species; NO, nitric oxide; ATP, adenosine triphosphate; OA, osteoarthritis; PBM, photobiomodulation. The black arrow represents the response induced by OA or inflammatory conditions, while the red arrow represents the efficacy of PBM.

This review summarizes the effects of laser radiation on OA modeling and inflammatory conditions in relation to cartilaginous tissue. Figure 1 shows the efficacy of PBM on OA and a set of possible mechanisms. Numerous animal- and cell-based studies have demonstrated the efficacy of PBMT for treating OA. The mechanisms of action include pain inhibition, suppression of inflammatory cytokine production, promotion of anti-inflammatory cytokine and ECM production, and suppression of ECM-destroying enzyme production. However, this study has some limitations and implications. First, although many studies have demonstrated the efficacy of PBMT, only a few have examined the effects of intracellular signals in chondrocytes in detail. According to our review of the literature, previous studies have only investigated the effects of PBMT on intracellular signaling pathways in chondrocytes. Studies examining the effects of PBMT on signaling pathways for cell types other than chondrocytes have reported effects on the MAPK/ERK [66], JNK/AP-1 [67], and Akt [68] signaling pathways, which may also have some impact on these signaling pathways, at least in chondrocytes. Further investigation of specific signaling pathways is warranted. Second, a very heterogeneous laser parameter was used in this study; therefore, rigorous comparison cannot be conducted between each study. The OA phenotype has also been studied extensively using OA modeling, but there have been a paucity of basic studies related to the effects of laser radiation on TMJ-OA. Compared with articular tissues of the knee, TMJ may differ in terms of the efficacy of laser irradiation and optimal irradiation conditions because of the different anatomy. Therefore, in-depth studies on the efficacy of PBMT using TMJ-OA modeling are necessary in the future. To establish parametric norms for distinct OA phenotypes, individual variations in tissue optical properties and energetic decay should be elucidated through systematic experimental designs, integrating biophotonic models, such as response-surface methodologies and simulations. Third, the existing animal studies (≤8 weeks) cannot assess the impact of PBMT on the structural progression of OA. Further long-term follow-up studies using large-animal models are necessary. Also, many studies have reported that the OA model is an inflammatory model that focuses only on local inflammation and changes in chondrometabolic markers and ECM. Therefore, pain is yet to be evaluated. To assess pain, assessment of nociceptive behavior and central inflammation, as well as pain markers such as Glial Fibrillary Acidic Protein, ionized calcium-binding adapter molecule 1, and c-fos in the trigeminal and sciatic ganglia may help to elucidate more detailed mechanisms. Fourth, OA not only involves chondrocytes but synovial fibroblasts, macrophages, and subchondral osteocytes are closely involved in OA. Additionally, chondrocytes have different properties in the superficial and middle layers of cartilage, and in subchondral osteocytes. Furthermore, the effects of PBMT on M1 macrophage-polarization and synovial HIF-1α signaling in cartilaginous tissues have not been elucidated. In addition, different cell types exhibiting mitochondria-specific response thresholds have not been studied in detail for differences in cytochrome c oxidase absorption peaks. Further studies on the effect of PBMT on the different cell types and these mechanisms are necessary. Fifth, reports from human studies have stated that PBMT is effective against OA [69,70,71], while others have stated that it is not [72,73,74,75,76]. This contradiction may be due to the difficulty in making simple comparisons due to different irradiation parameters (wavelength, number of irradiation sessions, duration of irradiation, and total energy). In the future, a high-quality prospective study using double-anonymized randomized controlled trials with an adequate sample size should be conducted in a multicenter setting to establish an irradiation protocol in which PBMT can be effectively and safely applied. PBMT is considered to be one of the most encouraging therapies in the treatment of OA because it is noninvasive, painless, and has no significant side effects reported to date. Nonetheless, scientific evidence strongly supporting the application of PBMT as a treatment for OA is lacking. Further clarification of the mechanisms underlying the effect of PBMT should be prioritized to promote PBMT as a therapeutic option for OA.

## Figures and Tables

**Table 2:** Summary of the effects of PBMT on inflammation models in in vitro studies.

Study ID	Author	Year	Irradiation Conditions	Cell Inflammation Model	Results
[41]	Chen et al.	2021	635 nm 2.74 mW 10.37 J/cm^2^ 2 h 1 session	Porcine chondrocytes H_2_O_2_	PBMT decreased free radical generation and IL-1β and TNF-α expression and promoted type II collagen expression.
[42]	Yang et al.	2020	632.8 nm 12 mW 5, 74 J/cm^2^ 8 min 5 sessions	Rabbit chondrocytes IL-1β	PBMT promoted cellular growth and cartilaginous matrix production and suppressed type I collagen and IL-1β expression and promoted type II collagen, aggrecan, CTNNB1, and SOX9 expression. On IL-1β stimulation, ADAMTS5, caspase-3, FADD, MMP-13, TNF-α, TNFR1, and TRADD expression was suppressed.
[43]	Hang et al.	2024	830 nm Continuous 0.056 W 5 J/cm^2^ Once every 2 days	Rabbit and human chondrocytes None	PBM increased relative intensity of collagen type II immunostaining and collagen type II expression in 2D culture. PBM and alginate-based scaffolds show promise for accelerating and optimizing cartilage regeneration, with potential application in tissue engineering.
[44]	Sakata et al.	2020	910 nm Pulsed 300 mW 8 J/cm^2^ 256 s 1 session	Human chondrocytes IL-1β	PBMT suppressed IL-1β, IL-6, TNF-α, MMP-1, and MMP-3 expression.
[45]	Sakata et al.	2022	910 nm Pulsed 300 mW 8 J/cm^2^ 256 s 1 session	Human chondrocytes IL-1β	PBMT suppressed NF-κB signaling, which was enhanced by IL-1β stimulation.
[46]	Tim et al.	2022	808 nm Continuous 50 mW 28, 50 J/cm^2^ 16, 25 s 5 sessions	Rat chondrocytes None	PBMT increased DNA content of chondrocytes.
[47]	Torricelli et al.	2001	780 nm Pulsed 2500 mW 10 min 5 sessions	Rabbit and human chondrocytes None	PBMT promoted cellular growth.
[48]	Anbari et al.	2024	808 nm Continuous 1, 2, 3, 4, 5 J/cm^2^	Human chondrocytes None	808 nm laser irradiation at energetic doses below 5 J/cm^2^ does not significantly increase chondrocyte proliferation.

## Data Availability

No new data were created or analyzed in this study. Data sharing is not applicable to this article.

## Abbreviations

The following abbreviations are used in this manuscript:

ACL	anterior cruciate ligament
ADSC	adipose-derived stem cell
CINC-1	chemoattractant-1
CS/Gl	chondroitin sulfate and glucosamine sulfate
ECM	extracellular matrix
IL	interleukin
LED	light-emitting diode
MIA	monoidoacetate
MMP	matrix metalloproteinase
MSC	mesenchymal stem cells
NIR	near-infrared
NO	nitric oxide
NSAID	non-steroidal anti-inflammatory drug
OA	osteoarthritis
OARSI	Osteoarthritis Research Society International
PBMT	photobiomodulation therapy
PGE2	prostaglandin E2
ROS	reactive oxygen species
TMJ	temporomandibular joint
